# High proportions of iron deficiency and thalassemia in blood donors with low RBC osmotic fragility: a call for targeted public health responses in Southern China

**DOI:** 10.3389/fpubh.2026.1804892

**Published:** 2026-03-19

**Authors:** L. Y. Sun, R. Li, D. H. He, Z. S. Xie, C. L. Liang, S. Q. Cai, J. F. Zeng, S. X. Wang

**Affiliations:** 1 Institution of Transfusion Medicine, Shenzhen Blood Center, Shenzhen, Guangdong, China; 2 Department of Laboratory Medicine, Shenzhen Blood Center, Shenzhen, Guangdong, China; 3 The Third People’s Hospital of Longgang, Clinical Institute of Shantou University Medical College (The Third People’s Hospital of Longgang District Shenzhen), Guangdong, China; 4 Department of Blood Transfusion, Shenzhen Third People’s Hospital, Shenzhen, Guangdong, China; 5 Department of Blood Transfusion, The Eighth Affiliated Hospital of Sun Yat-sen University, Shenzhen, Guangdong, China

**Keywords:** blood donation, iron deficiency, public health policy, thalassemia, transfusion safety

## Abstract

**Objective:**

This study aimed to investigate the prevalence and etiological profile of decreased red blood cell osmotic fragility among voluntary blood donors in Shenzhen, China, and to assess the public health implications for donor screening policies and transfusion safety in thalassemia-endemic regions.

**Methods:**

This cross-sectional study enrolled 362 voluntary blood donors at the Shenzhen Blood Center from July to September 2025. All donors underwent initial hemoglobin screening and red blood cell osmotic fragility testing. Donors with decreased fragility received comprehensive evaluation including complete blood count, serum ferritin measurement, hemoglobin electrophoresis, and thalassemia gene detection. Transfusion efficacy was preliminarily assessed by analyzing recipient hemoglobin increments.

**Results:**

Among the 14 donors (3.87%) with decreased red blood cell osmotic fragility, 9 (64.3%) were confirmed as thalassemia gene carriers (7 with *α*-thalassemia, 1 with *β*-thalassemia, and 1 with compound α/β-thalassemia), and 7 (50.0%) had biochemical iron deficiency (ferritin <30 ng/mL), with 4 individuals meeting both criteria, while 2 donors had neither thalassemia nor iron deficiency. Blood components from donors with decreased osmotic fragility, including those from thalassemia carriers, demonstrated effective hemoglobin recovery in recipients, with post-transfusion increments observed in all transfused cases.

**Conclusion:**

In Southern China, decreased red blood cell osmotic fragility among blood donors is primarily associated with thalassemia trait and iron deficiency. These findings highlight the need for targeted screening strategies and evidence-based policies to optimize donor health management while ensuring blood safety and availability. The study provides a public health framework for managing subclinical hematological conditions in blood donor populations in endemic regions.

## 1 Introduction

Voluntary blood donation systems represent a critical intersection of individual altruism and public health infrastructure. In regions with high prevalence of inherited hemoglobin disorders such as Southern China, blood services face unique challenges in balancing donor health protection, blood safety, and sustainable blood supply. While current donor screening protocols worldwide primarily focus on transmissible infectious diseases (1–3), routine hematological assessment is often limited to hemoglobin (Hb) or hematocrit (Hct) levels to prevent anemia in donors (4, 5). However, this approach may overlook subclinical hematological conditions that do not immediately manifest as overt anemia but could have implications for both donor health and, potentially, the quality of blood components. Two such prevalent conditions are thalassemia trait and iron deficiency.

The osmotic fragility test measures red blood cell hemolysis across a range of hypotonic sodium chloride concentrations and has long been employed to assess erythrocyte resistance to osmotic stress (6, 7). Reduced osmotic fragility is primarily attributed to an increased surface-area-to-volume ratio of red blood cells (8–10). This morphological feature is characteristic of iron deficiency anemia and thalassemia, both of which involve impaired hemoglobin synthesis resulting in reduced mean cell hemoglobin (MCH) and mean cell volume (MCV). These underfilled erythrocytes exhibit heightened resistance to osmotic lysis (11–14). In regions with a high prevalence of these conditions, a simple one-tube osmotic fragility test can serve as a practical screening tool for detecting thalassemia or iron deficiency (15–17).

Thalassemia trait is a common inherited hemoglobinopathy in Southern China (18, 19), while iron deficiency frequently results from uncompensated iron loss due to phlebotomy, particularly among frequent blood donors (20–22). Both conditions lead to microcytosis and hypochromia. Investigating RBC osmotic fragility in donor populations thus offers an integrative and novel perspective on subclinical hematological health.

The public health significance extends beyond donor health to blood transfusion outcomes. In resource-limited settings, questions persist regarding the appropriate management of blood components from asymptomatic thalassemia carriers. While current regulations in many countries do not prohibit donation by carriers, evidence-based guidelines for their optimal use remain limited.

Therefore, this study aimed to investigate the specific etiologies underlying decreased RBC osmotic fragility in a cohort of voluntary blood donors in Shenzhen. Using a structured diagnostic algorithm, including CBC, serum ferritin, hemoglobin electrophoresis, and thalassemia genotyping applied to donors preselected by osmotic fragility, we sought to determine the prevalence of thalassemia trait and iron deficiency in this subgroup. Additionally, we conducted a preliminary assessment of the transfusion efficacy of RBC units from these donors by tracking corresponding recipient hemoglobin increments post-transfusion. Our goal was to evaluate the utility of osmotic fragility as a donor health screening tool and to contribute evidence regarding the clinical suitability of blood from donors with these subclinical hematological abnormalities.

## 2 Materials and methods

### 2.1 Study population and sample collection

This cross-sectional study was conducted at the Shenzhen Blood Center from July to September 2025. A total of 363 consecutive voluntary blood donors, who met the standard national blood donation eligibility criteria, were enrolled. After blood donation, a 10-mL whole blood sample was collected from each donor into separate EDTA- and heparin-anticoagulated tubes. These samples were used for routine hematological testing as well as for subsequent specialized assays. The study protocol was reviewed and approved by the Ethics Committee of the Shenzhen Blood Center (Approval No. SZBCMEC-2025-026). Written informed consent was obtained from all participants prior to sample collection.

### 2.2 Initial screening: hemoglobin screening and red blood cell osmotic fragility test

Hemoglobin Screening by Automated Spectrophotometric Method: Donor hemoglobin eligibility was first assessed using a fully automated spectrophotometric method. Briefly, 30 μL of well-mixed EDTA-anticoagulated whole blood was added to 300 μL of specific hemoglobin lysing reagent (Xiwang Medical Devices Co., Ltd., Jinan, Shandong, China) to lyse red blood cells and release hemoglobin. The mixture was then analyzed on a fully automated biochemical analyzer (Xiwang Medical Devices Co., Ltd., Jinan, Shandong, China). Donors were deemed eligible if their hemoglobin concentration met or exceeded the national standard thresholds (≥125 g/L for males and ≥115 g/L for females). Only donors who passed this preliminary hemoglobin screen proceeded to standard whole blood donation and were included in the subsequent osmotic fragility testing.

The red blood cell (RBC) osmotic fragility test was performed using a modified one-tube method. Briefly, 30 μL of well-mixed EDTA-anticoagulated whole blood was added to 1 mL of isotonic saline (0.9% NaCl) in a test tube and thoroughly mixed by gentle inversion. The mixture was then analyzed on a fully automated biochemical analyzer (Beckman Coulter AU680, United States) within 10 min of preparation. Decreased osmotic fragility was defined as a result below the established reference threshold for our laboratory. Erythrocyte osmotic fragility was considered decreased when the hemolysis rate was less than 65%, based on the reference range according to the instructions of the reagents.

### 2.3 Extended diagnostic work-up for donors with decreased fragility

Donors identified with decreased RBC osmotic fragility underwent a comprehensive secondary evaluation.

#### 2.3.1 Complete blood count

A CBC was performed for all donors with decreased fragility using a Sysmex XN-9000 automated hematology analyzer (Sysmex Corporation, Japan) and its accompanying reagents. Parameters including hemoglobin (Hb), mean corpuscular volume (MCV), mean corpuscular hemoglobin (MCH) were recorded. Normal reference values are established for key erythrocyte indices: hemoglobin (Hb) levels are 130–175 g/L in males and 115–150 g/L in females, mean corpuscular volume (MCV) ranges from 82 to 100 fL, and mean corpuscular hemoglobin (MCH) from 27 to 34 pg.

#### 2.3.2 Serum ferritin measurement

The quantification of ferritin in human serum or plasma (heparin) was performed using a two-site immunoenzymatic (“sandwich”) assay on a Beckman DXI 800 automated immunoassay analyzer. Iron deficiency was defined as a serum ferritin level < 30 ng/mL (23), in accordance with clinical guidelines.

#### 2.3.3 Hemoglobin electrophoresis

Hemoglobin analysis was performed using capillary electrophoresis on a Sebia CAPILLARYS 3 OCTA system (Sebia, France) with the manufacturer’s accompanying reagents. Fresh EDTA-anticoagulated whole blood samples were loaded directly onto the sample rack for automatic analysis. The percentages of HbA, HbA_2_, and HbF were quantified (HbA 97.0–98.5%, HbA_2_ 2.5–3.0%, HbF 0%, and no abnormal hemoglobin bands).

#### 2.3.4 Thalassemia gene detection

Genomic DNA was extracted from peripheral blood leukocytes. Thalassemia gene detection was performed using a PCR-reverse dot blot hybridization kit (Shenzhen Yaneng Bio, China), which detects common *α*-thalassemia deletions (e.g., -^SEA^, -α^3.7^, -α^4.2^) and *β*-thalassemia point mutations (e.g., CD41-42, IVS-II-654, -28). A donor was confirmed as a carrier if one or more pathogenic mutations were identified.

### 2.4 Assessment of transfusion efficacy in recipients

For a subset of RBC components from study donors that were transfused, recipient data were retrieved from hospital databases. The primary outcome was the hemoglobin increment, calculated as the difference between post-transfusion (approximately 24 h after transfusion) and pre-transfusion (within 24 h before transfusion) hemoglobin levels. Recipient demographics and transfusion volume were also recorded.

## 3 Results

### 3.1 Initial screening: hemoglobin screening and red blood cell osmotic fragility test

A total of 363 blood donors with complete data were included in the final analysis. The cohort consisted of 210 male donors (57.9%) and 153 female donors (42.1%), with an age range of 19 to 60 years. The pre-donation hemoglobin levels varied from 115 g/L to 179.7 g/L.

Erythrocyte osmotic fragility values spanned a wide range, from 49 to 96%. Notably, 14 donors (3.9% of the cohort) exhibited osmotic fragility below the 65% threshold, which is defined as decreased osmotic fragility. Among these 14 donors, 8 were male (3.8% of all males) and 6 were female (3.9% of all females).

### 3.2 Results of comprehensive evaluation for donors with reduced osmotic fragility

The comprehensive hematological and biochemical evaluation of the 14 donors with decreased osmotic fragility (<65%) revealed distinct patterns.

#### 3.2.1 Post-donation hemoglobin and red blood cell indices

Post-donation hemoglobin (Hb) levels ranged from 103.0 g/L to 168.0 g/L. Strikingly, the majority of these donors (12 out of 14, 85.7%) exhibited microcytic hypochromic red blood cell indices. Specifically, the Mean Corpuscular Volume (MCV) was below the normal reference range (82–100 fL) in 12 donors, with values ranging from 67.1 fL to 72.6 fL. Similarly, the Mean Corpuscular Hemoglobin (MCH) was subnormal (<27 pg) in 12 donors, ranging from 20.7 pg. to 26.4 pg. Donors 5, 7, 8, and 9 presented with MCV and/or MCH within or near the normal range (Table 1).

**Table 1:** The results of initial screening.

Donor ID	Gender	Age	Hb before blood donation (g/L)	RBC osmotic fragility (%)
1	Male	50	136.1	53
2	Male	50	140.3	50
3	Male	28	125.9	56
4	Male	60	136.3	54
5	Male	38	151.6	61
6	Male	24	141.1	59
7	Male	36	170	62
8	Male	36	154.2	62
9	Male	47	137.7	49
10	Female	51	124.6	63
11	Female	38	132.5	53
12	Female	28	117.0	64
13	Female	54	130.6	58
14	Female	53	127.5	53

Hb before blood donation: Hemoglobin screening by automated spectrophotometric method before donation.

#### 3.2.2 Iron status and hemoglobin electrophoresis

Serum ferritin levels showed marked heterogeneity, spanning from deficient (4.7 ng/mL) to elevated (399 ng/mL). Hemoglobin electrophoresis results were predominantly normal for most donors. The HbA fraction was >97% in 10 donors. The HbA_2_ level was within the normal range (<3.5%) for 10 donors, while it was mildly elevated (5.6 and 5.9%) in donors 3 and 4, respectively. A slightly elevated HbF (>2%) was observed in donors 3, 4, and 11. No abnormal hemoglobin bands were detected in any donor.

Based on the comprehensive phenotypic screening, the 14 donors were categorized into four distinct groups as follows: (1) Probable *β*-thalassemia trait (*n* = 2): Donors 3 and 4; (2) Suspected iron deficiency (*n* = 7): Donors 4, 5, 8, 10, 11, 12, 14; (3) Normal hematological indices (*n* = 2): Donor 7 and 9; and (4) Phenotype suggestive of *α*-thalassemia trait (*n* = 4): Donors 1, 2, 6, and 13. Of note, Donor 4 exhibited features of both probable *β*-thalassemia trait (elevated HbA_2_, microcytosis) and suspected iron deficiency (low ferritin), illustrating the coexistence of these conditions. Therefore, this donor is included in both groups in the phenotypic classification (Table 2).

**Table 2:** Results of the comprehensive evaluation for donors with reduced fragility presented herein.

Donor ID	Hb after blood donation (g/L)	MCV (fL)	MCH (pg)	Serum ferritin (ng/mL)	Hemoglobin electrophoresis results			
					HbA (%)	HbA_2_ (%)	HbF (%)	Abnormal hemoglobin band (%)
1	110.0	70.0	21.0	45.7	97.5	2.5	/	/
2	116.0	72.1	21.7	116.2	97.4	2.6	/	/
3	103.0	69.6	21.2	92	90.9	5.6	3.5	/
4	136.0	67.7	21.3	9.71	93.8	5.9	0.3	/
5	141.0	85.7	28.2	5.77	97.4	2.3	0.3	/
6	131.0	68.9	21.1	399	97.7	2.3	/	/
7	168.0	94.0	31.9	158.1	97.5	2.5	/	/
8	148.0	87.9	29.9	21.2	97.5	2.5	/	/
9	131.0	95.9	32.4	131	97.4	2.6	/	/
10	127.0	82.1	26.4	5.16	97.7	2.3	/	/
11	106.0	67.1	20.7	4.7	97.1	2.5	0.4	/
12	108.0	71.4	21.5	22	97.5	2.2	0.3	/
13	130.0	67.6	20.9	62.4	97.8	2.2	/	/
14	110.0	72.6	23.0	11.5	97.6	2.4	/	/

“/” indicates not detected or not applicable. Hb after blood donation: Hemoglobin measured by complete blood count after donation.

### 3.3 Results of thalassemia genetic testing

Genetic testing identified thalassemia carriers among 9 of the 14 donors (Table 3). Among the seven *α*-thalassemia carriers, the -^SEA^ deletion was the most common (6/7, 85.7%), followed by the -ɑ^3.7^ deletion (1/7, 14.3%). No -ɑ^4.2^ deletion was detected in this cohort. The single *β*-thalassemia carrier harbored the IVS-II-654 mutation, and the compound carrier had both -ɑ^3.7^ and CD41-42 mutations.

**Table 3:** Genetic results of 14 donors.

Donor ID	α-Thalassemia gene detection	β-Thalassemia gene detection
1	Heterozygous for SEA-type deletion (Southeast Asian deletion)	/
2	Heterozygous for SEA-type deletion (Southeast Asian deletion)	/
3	/	Heterozygous for IVS-II-654 (C → T) mutation
4	Heterozygous for -α^3^·^7^ deletion	Heterozygous for CD41-42 (-TTCT) mutation
5	/	/
6	Heterozygous for SEA-type deletion (Southeast Asian deletion)	/
7	/	/
8	/	/
9	/	/
10	/	/
11	Heterozygous for SEA-type deletion (Southeast Asian deletion)	/
12	Heterozygous for SEA-type deletion (Southeast Asian deletion)	/
13	Heterozygous for SEA-type deletion (Southeast Asian deletion)	/
14	Heterozygous for -α^3^·^7^ deletion	/

The results for the other donors (indicated by “/” in the table) were negative for the common mutations tested in this screening.

Overlap analysis revealed that four donors (Donors 4, 11, 12, and 14) were both thalassemia carriers and iron deficient. Thus, among the 14 donors, 5 were thalassemia carriers only, 3 had isolated iron deficiency, 4 had both conditions, and 2 had normal hematological profiles.

### 3.4 Transfusion efficacy for recipients of blood from donors with decreased osmotic fragility

Based on the data presented in Table 4, red blood cell components from 5 of the 14 donors with decreased osmotic fragility were transfused to clinical recipients, with complete pre- and post-transfusion hemoglobin data successfully traced. Among these five donors, three (Donors 2, 4, and 12) were identified as thalassemia carriers. The underlying diseases in these recipients included abnormal uterine bleeding, gastrointestinal bleeding, acute lymphoblastic leukemia (ALL), severe β-thalassemia, and acute myeloid leukemia (AML). The volume of RBC units transfused ranged from 200 mL to 400 mL. All five patients showed increased hemoglobin levels at 24 h post-transfusion compared to pre-transfusion levels, with increments of 30 g/L (Donor 2), 11 g/L (Donor 4), 7 g/L (Donor 5), 7 g/L (Donor 10), and 5 g/L (Donor 12), respectively, representing a mean increase of 12 g/L. These findings indicate that RBC components derived from donors with decreased osmotic fragility, including those from thalassemia carriers, maintain satisfactory transfusion efficacy and can effectively elevate hemoglobin levels in recipients.

**Table 4:** Transfusion efficacy for recipients of blood from donors with decreased osmotic fragility.

Donor ID	Volume	Hb before transfusion	Hb after transfusion	Recipient information		
				Age	Gender	Clinical diagnosis
2	400 mL	42	72	36	Female	Abnormal uterine bleeding
4	400 mL	52	63	50	Male	Gastrointestinal bleeding
5	200 mL	53	60	27	Male	ALL
10	400 mL	69	76	13	Female	Severe β-thalassemia
12	400 mL	60	65	60	Male	AML

## 4 Discussion

This study identified thalassemia trait and iron deficiency as the primary factors associated with decreased RBC osmotic fragility among blood donors in Shenzhen. Of the 14 donors with decreased fragility, 9 (64.3%) were thalassemia carriers and 7 (50.0%) had iron deficiency, with some donors presenting both conditions (four donors in this cohort). Given these underlying hematological traits, monitoring hemoglobin (Hb) dynamics before and after donation offers valuable insights for donor health assessment and informs evidence-based donation policies. In this cohort, pre-donation Hb levels, measured via an automated spectrophotometric method, fell within the eligible range for donation (117.0–170.0 g/L). Following a standard 400 mL whole blood donation, post-donation Hb values obtained through complete blood count showed an average decline of approximately 8.7 g/L, which is consistent with expected physiological changes after phlebotomy.

Notably, post-donation Hb measurement is not merely an outcome metric but also a dynamic indicator of a donor’s erythrocyte reserve and iron status. Even among donors who exhibited underlying microcytosis and hypochromia, which suggested the presence of thalassemia trait or iron deficiency, post-donation hemoglobin levels remained above the symptomatic anemia threshold in nearly all cases. This indicates that individuals with subclinical hematological abnormalities can generally tolerate acute blood loss without immediate clinical compromise.

These findings support the potential utility of incorporating post-donation Hb monitoring into donor health surveillance systems. Such data could help identify donors who may benefit from extended inter-donation intervals or individualized iron supplementation, particularly in regions with high prevalence of thalassemia or iron deficiency. Furthermore, tracking post-donation Hb trends at a population level may inform the development of more nuanced donor eligibility criteria and personalized donation frequencies, thereby aligning donor safety with sustainable blood supply management.

In parallel, advancing the detection and understanding of specific underlying conditions, such as thalassemia, is essential for refining donor screening protocols. Thalassemia syndromes are recessively inherited hemoglobinopathies characterized by reduced or absent synthesis of hemoglobin chains, leading to chronic anemia of varying severity (24). In our cohort, hemoglobin electrophoresis effectively identified the two *β*-thalassemia carriers by elevated HbA_2_ levels but showed limited sensitivity for *α*-thalassemia, which typically presents with normal electrophoretic patterns. This underscores the necessity of combining phenotypic screening with genetic testing for comprehensive thalassemia identification in donor populations (25–27). Our genetic testing identified 9 thalassemia carriers included 7 *α*-thalassemia carriers, 1 isolated *β*-thalassemia carrier, and 1 compound α/β-thalassemia carrier. These findings indicate that *α*-thalassemia is more common than *β*-thalassemia in Guangdong Province, which is consistent with other research (28, 29). Our finding that -^SEA^ is the predominant *α*-thalassemia mutation is consistent with large-scale epidemiological studies in Guangdong Province (30), which also reported -^SEA^ as the most frequent deletion, followed by -α^3.7^ and -α^4.2^.

Moreover, in regions with high thalassemia prevalence, questions remain regarding the clinical utility and safety of blood components from asymptomatic carriers. Although such units exhibit altered RBC indices and possibly reduced osmotic fragility, their ability to raise hemoglobin levels in recipients and their overall impact on transfusion outcomes has not been extensively studied in systematic donor-recipient paired analyses. Our findings align with limited existing reports suggesting that thalassemia trait blood is hemodynamically effective and safe for transfusion (31). Nevertheless, in regions like Southern China with a high prevalence of thalassemia, blood services might consider implementing selective screening and labeling strategies for such units, enabling clinicians to make informed decisions based on recipient profiles, for example, preferring standard units for patients requiring chronic transfusion therapy, while utilizing carrier units in acute hemorrhage or surgery where immediate oxygen delivery is prioritized.

The preliminary follow-up of recipients receiving blood components from donors with thalassemia traits offers clinically relevant insights. Despite the donors’ hematological abnormalities characterized by microcytosis, hypochromia, and in some cases reduced osmotic fragility, their red blood cells were still capable of raising hemoglobin levels in recipients post-transfusion. This indicates that thalassemia trait donor blood retains functional oxygen-carrying capacity and can contribute effectively to acute hemoglobin recovery in compatible recipients.

Future studies with larger sample sizes, longer follow-up, and detailed *in vivo* red cell survival assessments are needed to further define the optimal use of blood from thalassemia carriers. Incorporating such evidence into transfusion guidelines would help harmonize donor health management with recipient clinical outcomes in thalassemia-endemic regions.

In addition to refining the use of blood from thalassemia carriers, systematic management of iron deficiency (ID) in donors represents another critical area for policy and practice improvement. Whole blood donors face an elevated risk of iron deficiency (ID) due to repeated phlebotomy-related iron loss (32, 33). In our cohort, iron deficiency was suspected in 7 out of 14 donors (50.0%) with reduced red cell osmotic fragility. Among these, four presented with microcytic hypochromic erythrocytes, while three showed normal red cell indices, consistent with early-stage or non-anemic iron deficiency. Although the association between iron deficiency and decreased red cell osmotic fragility is less frequently documented in the literature, our findings suggest that iron depletion may alter erythrocyte membrane properties and deformability even before overt anemia develops. For frequent donors, uncompensated iron loss from repeated donations represents a key risk factor, underscoring the importance of regular ferritin monitoring in this group. Without appropriate iron surveillance and supplementation, recurrent donation can pose significant health risks to donors. Our results therefore support the implementation of routine ferritin screening and individualized donation intervals based on iron status—an approach that has been shown to reduce iron deficiency in donor populations (34).

## 5 Conclusion

In summary, decreased RBC osmotic fragility in Shenzhen blood donors is strongly associated with thalassemia trait and iron deficiency. These conditions, while not contraindicating donation, necessitate enhanced monitoring and management strategies to protect donor health. The effective hemoglobin recovery observed in recipients of carrier blood supports its continued use, with appropriate clinical guidance. This study provides a public health framework for optimizing donor screening policies and blood management practices in regions with high thalassemia prevalence, balancing individual health protection with community blood supply needs.

## Data Availability

The original contributions presented in the study are included in the article/supplementary material, further inquiries can be directed to the corresponding authors.
